# Supplementary material for: Environmental DNA metabarcoding for monitoring metazoan biodiversity in Antarctic nearshore ecosystems
Source: PeerJ. 2021 Nov 15;9:e12458. doi: 10.7717/peerj.12458 (PMC8601059; doi:10.7717/peerj.12458)
Supplement: Supplemental Information 1 — Asterisks for eDNA locations indicate locations where sediment was processed from the same site/s. [file peerj-09-12458-s001.docx]

**Table S1.** Summary of nearshore eDNA and sediment samples processed for genetic biodiversity assessment. Asterisks for eDNA locations indicate locations where sediment was processed from the same site/s.

|  | **Water eDNA** | |  |  |  | **Sediment** |  |
| --- | --- | --- | --- | --- | --- | --- | --- |
| **Locations** | **No. sites** | **No. additional replicates** | **No. blanks** | **No. samples** |  | **No. sites** | **No. sections** |
| Abatus Bay* | 1 | - | - | 1 |  | 1 | 1 |
| Anchorage* | 3 | - | - | 3 |  | 2 | 10 |
| Bandits | 2 | 2 | 1 | 5 |  | - | - |
| Ellis Fjord | 4 | 2 | - | 6 |  | - | - |
| Hawker Channel | 2 | 1 | - | 3 |  | - | - |
| Powell Point east | 2 | - | - | 2 |  | - | - |
| Shirokaya Bay | 2 | 2 | - | 4 |  | - | - |
| STP9 | 2 | - | - | 2 |  | - | - |
| Warriner Channel* | 2 | 1 | - | 3 |  | 2 | 9 |
| Weddell Arm* | 3 | 1 | - | 4 |  | 2 | 10 |
| West Bay* | 2 | - | - | 2 |  | 2 | 10 |
| Wharf* | 1 | - | - | 1 |  | 1 | 2 |
| Lab blank | - | - | 2 | 2 |  | - | - |
| Totals | 26 | 9 | 3 | 38 |  | 10 | 42 |
