# Supplementary figures and images for: Environmental DNA metabarcoding for monitoring metazoan biodiversity in Antarctic nearshore ecosystems
Source: PeerJ. 2021 Nov 15;9:e12458. doi: 10.7717/peerj.12458 (PMC8601059; doi:10.7717/peerj.12458)

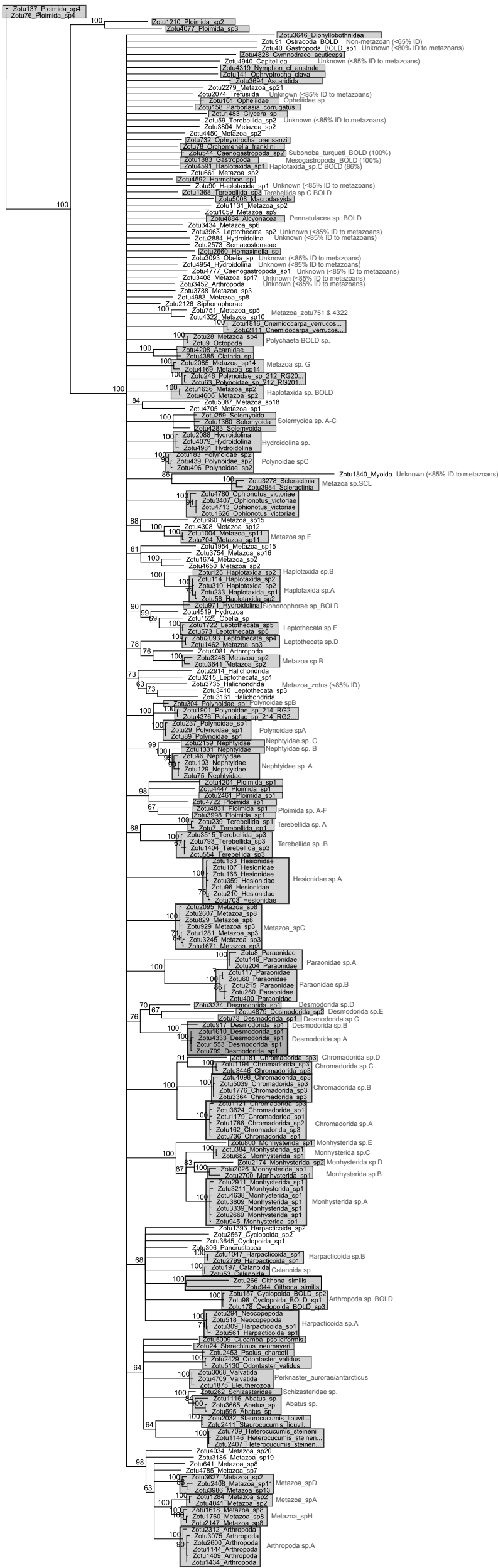

Supplement: Supplemental Information 2 — Taxonomic assignments that differed from the original assignment based on the BLAST search are also shown. [file peerj-09-12458-s002.pdf]

**A. Binary Jaccard**

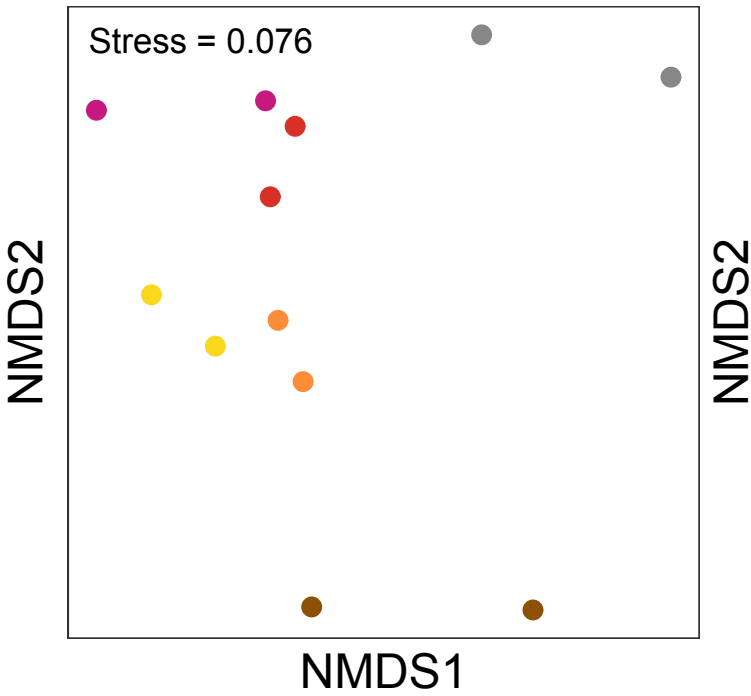

**B. Bray-Curtis**

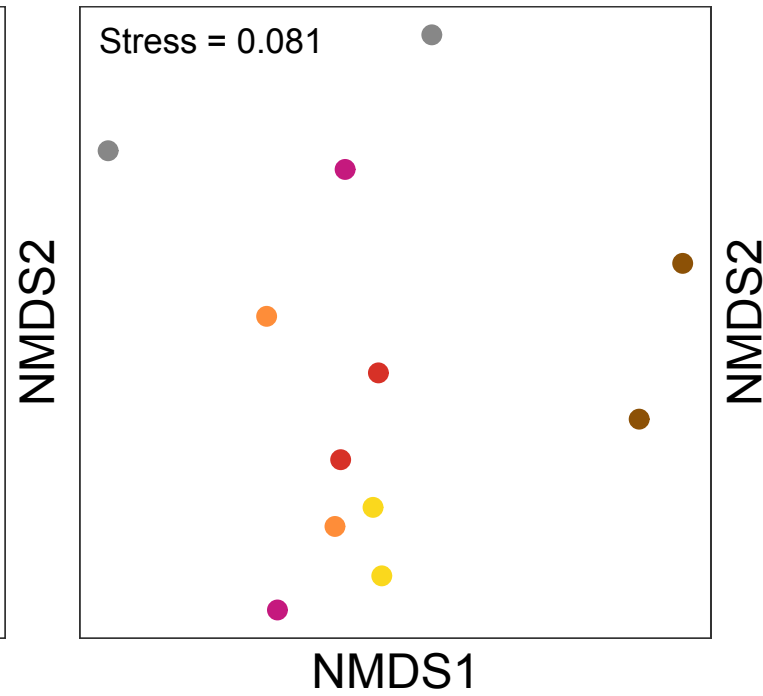

**C. Bray-Curtis, square-root transform**

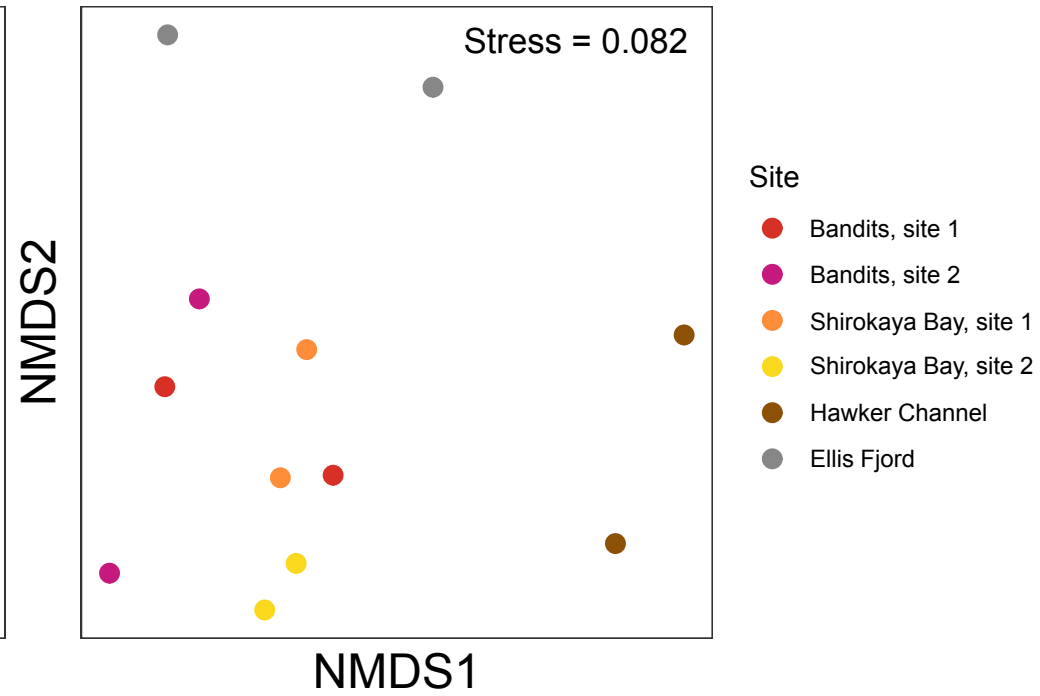

Supplement: Supplemental Information 3 [file peerj-09-12458-s003.pdf]
