# Supplementary material for: Environmental DNA metabarcoding for monitoring metazoan biodiversity in Antarctic nearshore ecosystems
Source: PeerJ. 2021 Nov 15;9:e12458. doi: 10.7717/peerj.12458 (PMC8601059; doi:10.7717/peerj.12458)
Supplement: Supplemental Information 5 [file peerj-09-12458-s005.docx]

**Environmental DNA metabarcoding for monitoring metazoan biodiversity in Antarctic nearshore ecosystems – Supplemental Information**

Laurence J. Clarke^1,2^, Leonie Suter^1^, Bruce E. Deagle^3^, Andrea M. Polanowski^1^, Aleks Terauds^1^, Glenn J. Johnstone^1^, Jonathan S. Stark^1^

***In silico* PCR**

We used *in silico* PCR to explore taxonomic coverage, resolving power and suitability of potential benthic arthropod metabarcodes, focussing on those targeting mitochondrial COI and 16S, and nuclear 18S rRNA genes. Target taxa were arthropods known to occur in East Antarctic benthic communities, molluscs and annelids (Table S2). To ensure primer-binding sites were present, we targeted complete or near-complete mitochondrial genomes, as well as complete 18S rRNA genes.

We searched the NCBI nucleotide database for mitochondrial genomes for each taxon in Table S1 by combining the search terms: “complete OR partial”, “mitochond*” and restricting the output to sequences between 10,000 and 20,000 bp. We searched the NCBI nucleotide database for complete 18S sequences for each taxon in Table S2 by combining the search terms: “complete AND 18S”, NOT “internal” (to remove internal transcribed spacer, or ITS, sequences) and restricting the output to sequences between 1,000 and 5,000 bp.

We used the program ECOPCR (Ficetola et al. 2010) to compare taxonomic coverage and resolution for each metabarcode in Table 1, S3 and S4. ECOPCR uses a pattern-matching algorithm to identify sequences within a database that can be amplified with a given primer pair by constraining the relative orientation of and maximum distance between primer-binding sites, as well as the number of mismatches between primer and target sequences (Ficetola et al. 2010).

Each sequence database (mitochondrial and complete 18S) was converted into a format compatible with ECOPCR using the ecoPCRFormat.py script. Inosine residues in the COI reverse primer (Table 1, S3 and S4) were changed to ‘N’. The ECOPCR parameters used were amplicon length between 50 and 1000 bp, with a maximum of three mismatches in each primer, with no restriction on mismatches in base pairs at the 3’ end. These settings are relaxed compared to *in vitro* PCR where mismatches in the last two base pairs at the 3’ end would prevent amplification, as the main question we were addressing was species-level resolution of the amplified sequence. The suitability of primer sequences for benthic arthropods should be checked and changed as necessary prior to their use in metabarcoding studies.

A caveat for *in silico* PCR with ECOPCR is that amplicons that differ by a single base are regarded as ‘resolved’. Greater sequence divergence would typically be desired to confidently resolve distinct taxa.

**Table S2.** DNA sequence data used for *in silico* PCR. Data downloaded from GenBank 10^th^ August 2021.

| **Phylum** | **Superorder/Class** | **Order** | **Mitochondrial genomes** | **Complete 18S** |
| --- | --- | --- | --- | --- |
| Arthropoda | Peracarida | Amphipoda | 181 | 120 |
|  |  | Isopoda | 37 | 88 |
|  |  | Tanaidacea | 1 | 1 |
|  |  | Cumacea | 1 | 1 |
|  | Ostracoda |  | 17 | 3 |
|  | Pycnogonida |  | 10 | 4 |
|  | *Total* |  | *247* | *217* |
|  |  |  |  |  |
| Annelida |  |  | 285 | 200 |
| Mollusca |  |  | 1907 | 407 |

**Table S3.** Primers, taxonomic coverage and resolution (species-level) of metabarcodes estimated by *in silico* PCR against a database of annelid mitochondrial genomes or complete 18S rRNA gene sequences.

| **Name** | **Locus** | **Primer sequence (5'-3')** | **Mean ± SD**  **length (bp)**  **(min.–max.)** | **Species-level coverage** | **Species-level resolution** | **Reference** |
| --- | --- | --- | --- | --- | --- | --- |
| Leray COI | COI | GGWACWGGWTGAACWGTWTAYCCYCC | 313 ± 1 | 112/119 (94%) | 112/112 (100%) | Leray et al. (2013) |
|  |  | TAIACYTCIGGRTGICCRAARAAYCA | (310-316) |  |  |  |
| Sauron COI | COI | GGDRCWGGWTGAACWGTWTAYCCNCC | 313 ± 1 | 115/119 (97%) | 113/115 (98%) | Rennstam Rubbmark et al. (2018) |
|  |  | TAIACYTCIGGRTGICCRAARAAYCA | (310-316) |  |  |  |
| Leray-XT | COI | GGWACWRGWTGRACWITITAYCCYCC | 313 ± 1 | 117/119 (98%) | 113/117 (97%) | Wangensteen et al. (2018) |
|  |  | TAIACYTCIGGRTGICCRAARAAYCA | (310-316) |  |  |  |
| V4_18S | 18S, V4 | CCAGCASCYGCGGTAATTCC | 379 ± 11 | 178/180 (99%) | 39/178 (22%) | Stoeck et al. (2010), |
|  |  | ACTTTCGTTCTTGATYRATGA | (376-508) |  |  | Piredda et al. (2017) |
| Euka03 | 18S, V9 | CCCTTTGTACACACCGCC | 138 ± 2 | 37/180 (21%) | 14/37 (38%) | Taberlet et al. (2018) |
|  |  | CTTCYGCAGGTTCACCTAC | (136-144) |  |  |  |
| Poly01 | 16C | CCGGTYTGAACTCAGMTCA | 63 ± 1 | 116/119 (97%) | 86/116 (74%) | Taberlet et al. (2018) |
|  |  | TGGCACCTCGATGTTGGCT | (61-66) |  |  |  |

**Table S4.** Primers, taxonomic coverage and resolution (species-level) of metabarcodes estimated by *in silico* PCR against a database of mollusc mitochondrial genomes or complete 18S rRNA gene sequences.

| **Name** | **Locus** | **Primer sequence (5'-3')** | **Mean ± SD**  **length (bp)**  **(min.–max.)** | **Species-level coverage** | **Species-level resolution** | **Reference** |
| --- | --- | --- | --- | --- | --- | --- |
| Leray COI | COI | GGWACWGGWTGAACWGTWTAYCCYCC | 313 ± 1 | 532/622 (86%) | 492/532 (92%) | Leray et al. (2013) |
|  |  | TAIACYTCIGGRTGICCRAARAAYCA | (307-313) |  |  |  |
| Sauron COI | COI | GGDRCWGGWTGAACWGTWTAYCCNCC | 312 ± 2 | 590/622 (95%) | 545/590 (92%) | Rennstam Rubbmark et al. (2018) |
|  |  | TAIACYTCIGGRTGICCRAARAAYCA | (304-319) |  |  |  |
| Leray-XT | COI | GGWACWRGWTGRACWITITAYCCYCC | 312 ± 2 | 604/622 (97%) | 559/604 (93%) | Wangensteen et al. (2018) |
|  |  | TAIACYTCIGGRTGICCRAARAAYCA | (304-319) |  |  |  |
| V4_18S | 18S, V4 | CCAGCASCYGCGGTAATTCC | 443 ± 114 | 321/326 (98%) | 231/321 (72%) | Stoeck et al. (2010), |
|  |  | ACTTTCGTTCTTGATYRATGA | (378-925) |  |  | Piredda et al. (2017) |
| Euka03 | 18S, V9 | CCCTTTGTACACACCGCC | 137 ± 9 | 82/326 (25%) | 53/82 (65%) | Taberlet et al. (2018) |
|  |  | CTTCYGCAGGTTCACCTAC | (120-192) |  |  |  |
| Gast01 | 16S | CCGGTCTGAACTCAGATCA | 64 ± 2 | 586/622 (94%) | 286/586 (49%) | Taberlet et al. (2018) |
|  |  | TTTGTGACCTCGATGTTGGA | (60-76) |  |  |  |

**Supplementary References**

Ficetola GF, Coissac E, Zundel S, Riaz T, Shehzad W, Bessière J, Taberlet P, and Pompanon F. 2010. An *in silico* approach for the evaluation of DNA barcodes. *BMC Genomics* 11:434. DOI: 10.1186/1471-2164-11-434

Leray M, Yang JY, Meyer CP, Mills SC, Agudelo N, Ranwez V, Boehm JT, and Machida RJ. 2013. A new versatile primer set targeting a short fragment of the mitochondrial COI region for metabarcoding metazoan diversity: application for characterizing coral reef fish gut contents. *Frontiers in Zoology* 10:34. DOI: 10.1186/1742-9994-10-34

Piredda R, Tomasino MP, D'Erchia AM, Manzari C, Pesole G, Montresor M, Kooistra WH, Sarno D, and Zingone A. 2017. Diversity and temporal patterns of planktonic protist assemblages at a Mediterranean Long Term Ecological Research site. *FEMS Microbiology Ecology* 93:fiw200. DOI: 10.1093/femsec/fiw200

Rennstam Rubbmark O, Sint D, Horngacher N, and Traugott M. 2018. A broadly applicable COI primer pair and an efficient single-tube amplicon library preparation protocol for metabarcoding. *Ecol Evol* 8:12335-12350. DOI: 10.1002/ece3.4520

Stoeck T, Bass D, Nebel M, Christen R, Jones MD, Breiner HW, and Richards TA. 2010. Multiple marker parallel tag environmental DNA sequencing reveals a highly complex eukaryotic community in marine anoxic water. *Molecular Ecology* 19 Suppl 1:21-31. DOI: 10.1111/j.1365-294X.2009.04480.x

Taberlet P, Bonin A, Zinger L, and Coissac E. 2018. *Environmental DNA: For biodiversity research and monitoring*. Oxford, UK: Oxford University Press.

Wangensteen OS, Palacin C, Guardiola M, and Turon X. 2018. DNA metabarcoding of littoral hard-bottom communities: high diversity and database gaps revealed by two molecular markers. *PeerJ* 6:e4705. DOI: 10.7717/peerj.4705
